# Finding the leaders: an examination of social network analysis and leadership identification in STEM education change

**DOI:** 10.1186/s40594-018-0124-5

**Published:** 2018-06-20

**Authors:** Alexis V. Knaub, Charles Henderson, Kathleen Quardokus Fisher

**Affiliations:** 1 Western Michigan University, Kalamazoo, MI USA; 2 Florida International University, Miami, FL USA

**Keywords:** Social network analysis, Higher education change, Higher education leadership

## Abstract

**Background:**

Social network analysis (SNA) literature suggests that leaders should be well connected and can be identified through network measurements. Other literature suggests that identifying leaders ideally involves multiple methods. However, it is unclear using SNA alone is sufficient for identifying leaders for higher education change initiatives. We used two sets of data, teaching discussion network data taken at three different times and respondent nominations for leaders, to determine whether these two methods identify the same individuals as leaders.

**Results:**

Respondent-nominated leaders have more direct and indirect ties on average than non-leaders, which aligns with the SNA literature. However, when looking at individuals as leaders, many respondent-nominated leaders would not be identified using SNA because they are poorly connected. Also, many individuals who were not nominated would have been considered leaders because they are well connected. Further examining these results did not indicate why there is such a difference between the SNA-identified and respondent-nominated leaders.

**Conclusions:**

While these two methods identify some of the same individuals as leaders, there are many differences between the two methods. Using just one method may not be adequate for ensuring that suitable individuals are selected to lead these projects. We recommend multiple methods when selecting leaders.

## Background

STEM change initiatives, multiple projects undertaken to broadly improve STEM education, have been part of the landscape to improve teaching practices in STEM higher education for decades. The success of these resource intensive endeavors relies on multiple individuals who are considered “champions,” enthusiastic persons who can lead efforts (e.g., Scherr et al. 2014). One suggested way to identify these leaders is using social network analysis (SNA) because it can provide a way to study the underlying, hidden structure of departments (Quardokus and Henderson 2015). However, it is unclear whether SNA data alone are adequate for identifying leaders. This paper builds on Quardokus and Henderson’s work by determining whether SNA data by itself is adequate for identifying leaders. The data were collected three times over 4 years at a single institution and analyzed to find differences in network position between leaders and non-leaders.

## Literature review

### Social network analysis and change in higher education

Researchers from a wide variety of disciplines (e.g., sociology, physics, public health) use social network analysis (SNA) to study relationships among entities (Wasserman and Faust 1994). Relationships can be any type of connection or tie including self-selected ties (e.g., friendships) or ties by circumstances (e.g., kinship, department membership). Entities can be people, groups, or organizations, and they can be referred to by multiple names. For ease of discussion, we use the term “actors” to refer to people within the network.

Much can be discovered from understanding relationships among actors. In K-12 education reform, network structure has been shown to influence change efforts through multiple mechanisms such as teachers providing advice and support to one another (Daly 2010; Coburn et al. 2012; Finnigan and Daly 2012; Penuel et al. 2012). In these networks, teachers having ties to each other contributed to the success of these change efforts.

There are many ways to study network structure. Network metrics consider both individual actors or the entire network. Common metrics include centralities (i.e., who is the most important actor in the network?), groups (e.g., how large and tightly connected is a group?), and density (i.e., how connected are the actors to one another within the network?). Metrics can also examine how the network changes over time. SNA metrics can be further analyzed using descriptive statistics (e.g., averages) and comparative statistics (e.g., *t* tests). Attributes of actors in the network can be useful for understanding how different identities can impact networks.

The attribute of interest for this study is leadership because of its critical role in groups. Teams with leaders who are central to team members tend to perform better (e.g., Balkundi and Harrison 2006). Leaders can also play a role in exchanging knowledge, and diffusing information between groups (Burt 1999) can be seen in STEM education. For example, Andrews et al. (2016) found that colleagues knowledgeable about education, such as discipline-based education research (DBER) faculty, are often considered to be opinion leaders by their peers who were interested in changing teaching practices.

Currently, SNA is not often used to study instructional practices or change in higher education. Biancani and McFarland’s (2013) study found only 117 SNA articles related to higher education faculty, with no articles on higher education faculty teaching networks and most on co-authorship or citation networks. Since the Biancani and McFarland article, a few articles that study higher education teaching networks have been published (e.g., Quardokus and Henderson 2015; Andrews et al. 2016). Despite the lack of current use, there have been some calls to use SNA to inform change initiatives (e.g., American Association for the Advancement of Science 2015).

### Changing teaching practices in STEM higher education and identifying those to lead efforts

#### Sustaining changed teaching practices in STEM higher education

Changing teaching practices is a social activity from learning about teaching innovations (Borrego et al. 2010; Dancy and Henderson 2010; Turpen et al. 2016) to implementing the new practice. Work such as American Association for the Advancement of Science’s (AAAS) *Vision and Change* (American Association for the Advancement of Science 2015) and Association of American Colleges and Universities (AAC&U)’s Project Kaleidoscope (Elrod and Kezar 2014) recommend creating coalitions of instructors and administrators to work on change initiatives. Although many STEM education change initiatives perceive individuals as the unit of change, these change initiatives are believed to be more successful when the entire department is involved (Wieman et al. 2010; Henderson et al. 2011; Zhu and Engels 2013; Foote et al. 2016) in order to gain support from multiple members.

#### Leadership identification in higher education change

Based on their work on Project Kaleidoscope, Elrod and Kezar (2014) posit that leadership is needed to create widespread change. These leaders act as champions, members of the organization who can garner support for an innovation and overcome challenges that may block adoption of the innovation (Rogers 2003). A successful champion or leader should have skills such as knowing how to create the conditions for change and be able to work with others when implementing change initiatives (e.g., Foote et al. 2016; Knaub et al. 2016).

Finding faculty who are willing to lead efforts can be challenging (American Association for the Advancement of Science 2015). While one could call upon the same individuals typically involved, this can create issues including few faculty are involved, which may limit change efforts (Wieman et al. 2010); individuals who are weary of being called upon (Rogers 2003); and potential leaders not being mentored by more senior leaders (e.g., Martin and Marion 2005). At the same time, simply selecting anyone to lead is not a solution. Not all individuals want to improve STEM education and may actively resist efforts to do so (Wieman et al. 2010; Foote et al. 2016).

Few studies exist in higher education regarding leadership identification. The ones that do tend to delve into formal leadership identification and not informal leadership (e.g., Bisbee’s (2007) exploratory study on administrative leaders such as deans). Because of this research gap, public health literature has informed this study. Public health seeks to change behaviors and uses a variety of techniques including individuals lead initiatives that encourage healthy behaviors (e.g., Kelly et al. 1991; Atkins et al. 2008; Kelly 2004). This is akin to how STEM education initiatives work to change teaching behaviors.

Public health research on programmatic efforts found that organizations identify leaders in many ways including role or title (e.g., an institution names a provost), self-nomination (e.g., individuals nominate themselves as leaders), nomination by members of the group (e.g., individuals nominate others), nomination by formal leaders or staff members working on the program, expert nomination (e.g., staff members nominate known experts in the area), and sociometric measures (e.g., using SNA to determine who is a leader based on some network metric) (Valente and Davis 1999; Valente and Pumpuang 2007).

In a review of literature on health-related leadership, Valente and Pumpuang (2007) found that each type of leadership selection method has pros and cons. For nomination methods, some of the pros include selecting individuals who are trusted by the targeted community and being fairly easy to implement. For nomination methods, some of the cons include selection bias (e.g., selecting only one’s friends regardless of suitability), leaders’ abilities may not be adequate if respondents only vaguely know those they select, volunteer leaders may not be capable of leading, and the nominated person may not be interested in leading. For sociometric methods, the pros include being able to select a leader based on their position in the social structure (e.g., selecting a leader who is highly connected) and typically being able to evaluate leaders based on multiple measures (e.g., selecting a leader who has many direct and indirect ties). The cons include that the methods can be time-consuming and are contingent upon who provides information on the network. They suggest that when possible, using multiple methods is ideal to identify leaders.

Although not ideal, perhaps using one method, such as SNA, is adequate for leadership identification. SNA literature implies that leaders may have networks that are different from non-leaders (Balkundi and Kilduff 2006; Watts and Dodds 2007; Katona et al. 2011). Some studies (e.g., Jonnalagadda et al. 2012; Xu et al. 2014) suggest that leaders can be identified through their metrics, such as centralities, and do not suggest using multiple methods.

Knowing whether SNA data alone are adequate for selecting a leader can better inform SNA use in this area. Given that using SNA to inform higher education change is relatively new but growing area, providing guidance to novices can support their success in using this tool which in turn can support their success in their change initiative. Departments engaged in STEM education change initiatives may already have SNA data and may plan on using these data to identify leaders. If SNA data are not adequate for selecting leaders, alternatives methods should be considered.

### Summary and research question

Although it is infrequently used in higher education change, SNA shows great potential to deepen our understanding in this area. Changing teaching practices in STEM higher education is a complex process involving the interactions of multiple people. SNA, which is used as a tool to describe social interactions, has a rich body of literature that indicates social interactions influence people’s behaviors.

Leadership identification is one possible way SNA data could be used. Having suitable leaders involved in higher education change who are interacting with others is important. In ideal circumstance, it is suggested that leadership identification is done using multiple methods. However, despite best efforts, ideal practices do not always occur. While triangulation may ideal, it is unclear how imperfect using one method is. In particular, it is unclear whether each method yields a different set of leaders or many of the same leaders.

Based on this ambiguity, our core research question asks whether SNA metrics alone be used to predict or identify recognized leaders in STEM education change?

## Methodology

### Sample description

The data in this manuscript are part of a large study of a single institution involved with a grant-funded STEM change initiative that began in 2012. The Carnegie classification of this institution is a large, 4-year primarily residential, very high research activity comprehensive doctoral institution. The change initiative features many projects that require social interactions: faculty learning communities, live-learn communities for students that involve multiple teaching staff members, and course reform. The change initiative originally involved five departments but expanded to six by the second year.

### Data collection

Social network data were initially collected as part of a long-term study to see if teaching discussion networks change as the change initiative projects matured and expanded. Data were collected at three different timestamps: spring 2012, spring 2013, and spring 2016.

A total of 315 unique actors appeared in at least one timestamp in the teaching discussion network data. Instructors (professors, lecturers, adjunct instructors, laboratory coordinators, and postdoctoral researchers) who taught during the academic year were invited to participate. Response rates are in Table 1. Spring 2012 marks the second year of a 4-year change initiative, and 2013 is the third year. The grant was renewed for an additional 4 years. The spring 2012 data were analyzed and featured in an article by Quardokus and Henderson (2015).

**Table 1:** Survey response rates by data collection timestamp

	Data collection no. 1		Data collection no. 2		Data collection no. 3	
	2011–2012		2012–2013		2015–2016	
Dept.	Individuals invited to survey	Response rate (%)	Individuals invited to survey	Response rate (%)	Individuals invited to survey	Response rate (%)
A	57	44	61	44	45	47
B	21	38	23	44	30	47
C	38	37	38	55	34	47
D	44	52	44	50	47	51
E	40	45	41	61	32	47
F	–	–	60	53	75	28

The first question of the survey, regardless of administration year, included a question asking respondents to select their teaching discussion partners from the academic year. “Teaching discussions” were not defined on the survey, but interview data from Quardokus and Henderson (2015) suggested that these discussions consisted of course or pedagogy issues as well as course logistics and staffing issues.

Respondents used a drop-down menu to select a maximum of seven teaching discussion partners from a roster. Limiting the number of discussion partners to seven was based on pilot data. The roster was based on the current teaching staff in the department. For timestamps 2 and 3, we also included individual who were not currently teaching but were listed on prior versions of the survey. This was to see if they continued to be active within teaching discussion, despite having no teaching responsibilities.

The 2016 version of the survey included additional questions related to leadership in STEM education change. Respondents were asked to identify current and potential leaders using a drop-down menu that contained the roster for their department, identical to the drop-down menu for teaching discussion. Respondents were given the opportunity to name additional individuals. We limited the number of responses to five for each type of leader. We did not define current or potential leaders, but we emphasized the prior success and importance of the change initiative to improve STEM education and the need to have individuals lead efforts. For the potential leader question, we emphasized the importance of fostering the next generation of leaders to take over when current leaders step down.

### Analysis

We treated the teaching discussion ties as undirected. For example, suppose individual A selected individual B on a survey. If these data were treated as undirected, it is assumed individual B also selected individual A, regardless of whether individual B did. For leadership identification, many SNA studies treated their data as directed, which means ties are not automatically mutual. Using the prior example, if the data were treated as directed, it is not automatically assumed individual B selected individual A. However, these studies also asked questions regarding who influences the respondent. An advice network would be an example of a directed network. Teaching discussion can be influential but is not necessarily directed.

The data presented only include respondents and actors that respondents selected. If a member of the department did not take the survey and was not named by a respondent, the member was not included in the network because their ties in the network are unknown.

**Fig. 1:**
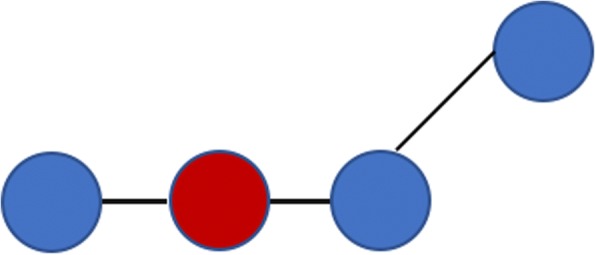
Example network consisting of four actors. The middle actor is shaded red and has a tie to each blue actor on the ends

All data were analyzed using UCINET (Borgatti et al. 2002) and SPSS 23. As we were interested individual behaviors, we used on the following metrics that focus on individual actors:

- *Degree centrality* counts the number of ties an actor has (Wasserman and Faust 1994). Someone with high degree centrality may be able to diffuse information more broadly than someone with low degree centrality. In Fig. 1, the red middle actor has two ties while each blue actor has one tie. For this study, degree centrality is the total number of ties an actor has.
- *Reach centrality* counts the number of actors that the ego, the actor of interest, can contact within *k* ties. This metric suggests the possibility of messages traveling between actors (Wasserman and Faust 1994). We anticipated that this would be an important measure because the ego’s ideas can travel indirectly to more actors. Figure 1, the red actor has a two-step reach of three because it has two direct ties plus one tie that can be reached through two steps.
  We used a specific type of reach centrality, two-step centrality. This can be thought of as an ego’s ties plus the friends of friends. While studies have indicated that behavioral influence can occur as far as three steps away (e.g., Christakis and Fowler 2007; Christakis and Fowler 2009), an ego is only aware of the activities of their direct ties and ties two steps away (e.g., friends of friends) (Friedkin 1983). Because those leading change initiatives should be knowledgeable of actors in their departments, we used two-step centrality and not 3-step centrality.

Because each year and department had different numbers of actors, we used *relative* measures for each centrality. Relative measures make comparison possible when the number of actors varies significantly from network to network (Scott 2012). Relative measures were found by dividing a measure of an individual actor by *n* - 1 where *n* is the total number of actors in the network. For example, suppose department *X* has 11 members. If one actor has six ties, relative degree centrality of this actor is 0.6.

As mentioned earlier, these metrics can be analyzed like other social science data. As we were interested in comparisons among groups (i.e., leaders and non-leaders), we used ANOVAs to determine if there were any statistically significant difference among groups. ANOVAs were used instead of *t* tests because as we will discuss in the next section, we had three categories. We used post hoc tests to determine which two groups had statistically significant differences. Post hoc Tukey tests were used when variance was equal and Games-Howell when it was not.

### Data categorization

On the survey, respondents indicated which actors they believed are current and potential leaders for STEM education reform. Based on the data, we refined these categories. As a reference, Table 2 displays succinct definitions.

**Table 2:** Definitions of leadership categories

Leader	Actor who has received 2+ nominations as a leader (current or potential)
Both leader	Actor who has received an almost equal number of nominations in the current and potential categories. The maximum absolute difference between the two categories is 1.
Current leader	Actor who has received 2+ nominations as a leader, with the majority of nominations as a current leader (2+ more nominations as a current leader than as a potential leader)
Potential leader	Actor who has received 2+ nominations as a leader, with the majority of nominations as a potential leader (2+ more nominations as a potential leader than as a current leader)
Maybe leader	Actor who has received only one nomination as a leader
Non-leader	Actor who has receive no nominations as a leader

Initially, we examined our data for two categories: *non-leaders* and *leaders*. Actors who were not nominated as leaders were considered non-leaders. We were interested whether actors were genuinely leaders and assumed that the more nominations an actor received, the more likely that an actor was truly a leader. However, we did not want to simply categorize actors who received few nominations as non-leaders and hypothesized they may be different from non-leaders and leaders. Looking at frequencies of leadership nominations, we found that the largest break was between 1 and 2 nominations. Thus, we categorized those with one nomination as *maybe leader* and those with 2+ nominations as leaders.

Our original categories, *current* and *potential*, also needed to be modified. Some individuals were only nominated within one category (e.g., respondents only selected actor A as a current leader), making it clear in which category the respondents thought the actor belonged. Many other actors were nominated at least once in each category, making it less clear in which category they belonged.

To determine in which category an actor belonged, we first found the absolute difference between these two categories. We subtracted the nominations for current leader from the nominations for a potential leader and took the absolute value. For example, if an actor received six nominations as a current leader and seven for a potential leader, the absolute difference would be 1. To create the *both* category, we looked at the frequency of absolute differences to determine where the largest break in the data was. Actors who were nominated equally for current and potential, as well as actors who had an absolute difference of 1 were placed in the both category. If the difference was greater than 1, actors were placed in the majority category. For example, an actor who was nominated three times as a potential leader and five times as a current leader would be considered a current leader.

### Limitations

The primary limitation of this study is the data may be incomplete. Departmental response rates range from 28 to 61% over the three timestamps (see Table 1). Departmental networks represent 46 to 87% of the possible actors. This means that some actors may not appear in the data even though they were involved in teaching discussion. For the 2016 version of this survey, there may be more leaders within each department but those who would nominate them did not take the survey.

Non-responsiveness is common with SNA using survey data (Žnidaršič et al. 2012; Morris and Deckro 2013). While there are various techniques (e.g., exponential random graph models, imputation methods that add ties to the network) to attempt to rectify this issue, there is currently no standard method for handling missing data (Stork and Richards 1992; Morris and Deckro 2013). Methods to rectify missing data can also introduce other issues as they are built on assumptions that ties would exist for non-respondents (Žnidaršič et al. 2012; Morris and Deckro 2013).

One suggested method for communication networks is to consider the data undirected (Stork and Richards 1992). This is the method we chose. The caveats are that the respondents and non-respondents should be similar in demographics and that the type of communication is undirected (e.g., discussion is undirected, advice may be directed) (Stork and Richards 1992). As seen in Table 1, respondents and non-respondents are present in each department.

To determine if there were any other demographic differences, we further examined the data to see how many respondents and non-respondents were involved with the change initiative. We used chi-square tests for each timestamp to determine whether there is a significant relationship between responding to the survey and being part of the change initiative. These data are displayed in Table 3. All chi-square results were found to have *p* > 0.05, thus response was not related to participating in the change initiative.

**Table 3:** Comparison of respondents and non-respondents

	*N* respondents involved	*N* of non-respondents involved	*N* of respondents not involved	*N* of non-respondents not involved	*x* ^*2*^
2011–2012	23	30	65	82	0.0107
2012–2013	31	22	106	108	1.36
2015–2016	26	26	85	126	1.6144

Another limitation is that this study focused on one institution engaged in change initiative activities that either directly (e.g., faculty learning communities) or indirectly (e.g., course reform) encourage faculty and staff to communicate. These findings may be different for institutions not engaged in a change initiative. Network metrics may also be different by institution type (e.g., a small liberal arts college). These data should be considered the beginning of this line of work; further research at both similar and different institutions is needed to determine what is typical and whether teaching discussion network metrics vary by institution type or other variables.

## Results

Three-hundred fifteen actors appear as a teaching discussion partner in at least one of the timestamps. Respondents nominated a total of 150 individuals (47.6% of all actors in the network) as leaders within their departments. The average number of times an individual was nominated as a leader is 4 ± 1.^1^ In four of the six departments, nine respondents nominated themselves as leaders. All self-identified leaders except for one were nominated multiple times by other respondents.

Using the definitions in Table 2, the sample breaks down as follows:

- Non-leaders (*N* = 165)
- Maybe leaders *(N* = 57)
- Leaders (*N* = 93)
  - Current leaders (*N* = 32)
  - Potential leaders (*N* = 29)
  - Both leaders (*N* = 32)

The leader category (*N* = 93 or 29.5% of all actors) consists of those nominated as a current leader or a potential leader at least twice. The maybe leader category (*N* = 57) consists of individuals nominated as a current or potential leader only once. Table 4 displays the breakdown of leaders and maybe leaders categories by department. The departments range from having 20 (department D) to 12 identified leaders (departments B and E).

**Table 4:** Descriptive statistics of individuals identified as leaders and maybe leaders by department

	Leader	% of roster	Maybe leader	% of roster
Dept. A	13	20	9	13.8
Dept. B	12	40	7	23
Dept. C	19	38.8	6	12.2
Dept. D	20	37	13	24.1
Dept. E	12	24	10	20
Dept. F	17	18.3	12	12.9

Department B has the highest percentage of nominated leaders (40%) while department F has the lowest (18.3%). Department F’s low percentage of leaders may be due to a lower response rate for the 2016 survey; we do not know why their response rate was lower than the other departments in 2016 or lower than their 2012 response rate. Department B’s response rate was comparable to the other departments. There may be other factors at play, such as department size (e.g., the department is small enough so that members are aware of their colleagues’ STEM education leadership activities) or department culture (e.g., the department encourages STEM education leadership).

We analyzed the data using ANOVAs to see if there were any differences among departments and the two centralities (degree and reach). There are no statistically significant differences *(p* < 0.05).

### Can SNA metrics be used to predict or identify recognized leaders in STEM education reform?

We initially analyzed the data to see if there were any differences between the three groups: leaders, maybe leaders, and non-leaders. Data were analyzed by timestamp, as we anticipated there might be differences in each timestamp due to the change initiative’s activities. Recall that the first data collection point in 2012 was near the beginning of the change initiative, while the grant had been active for several years by the third data collection point in 2016. Table 5 displays these results. ANOVAs reveal that the average values for degree and reach centralities are statistically significant (*p* < 0.05) among the groups. The results from the post hoc tests indicate that the difference between leaders, and the other two groups are statistically significant for *p* < 0.05.

**Table 5:** Social network metrics *t* test results comparing leaders, possible leaders, and non-leaders

			Leaders	Maybe Leaders	Non-Leaders
Data collection no. 1	2012	Degree centrality *F*(2, 57) = 8.30*	0.18 ± 0.02 (*N* = 42)	0.09 ± 0.02 (*N* = 24)	0.1 ± 0.009 (*N* = 75)
		Reach centrality *F*(2, 138) = 7.75*	0.48 ± 0.04 (*N* = 42)	0.30 ± 0.04 (*N* = 24)	0.31 ± 0.03 (*N* = 75)
Data collection no. 2	2013	Degree centrality *F*(2, 72) = 26.7*	0.19 ± 0.14 (*N* = 61)	0.1 ± 0.01 (*N* = 31)	0.08 ± 0.007 (*N* = 134)
		Reach centrality *F*(2, 223) = 23.5*	0.56 ± 0.03 (*N* = 61)	0.38 ± 0.04 (*N* = 31)	0.32 ± 0.02 (*N* = 134)
Data collection no. 3	2016	Degree centrality *F*(2, 106) = 22.8*	0.17 ± 0.01 (*N* = 83)	0.1 ± 0.01 (*N* = 42)	0.08 ± 0.007 (*N* = 82)
		Reach centrality *F*(2, 204) = 25.3*	0.54 ± 0.02 (*N* = 83)	0.41 ± 0.03 (*N* = 42)	0.33 ± 0.02 (*N* = 82)
	Longevity	Presence in network at least once at each timestamp (max. 3) *F*(2, 128) = 2.65	2 ± 1 (*N* = 93)	2 ± 1 (*N* = 165)	2 ± 1 (*N* = 57)

*p < 0.05

We were also interested in whether actors appear in all three timestamps, regardless of how many ties they have. Our hypothesis was that leaders would appear in more timestamps than maybe leaders and non-leaders and that simply being present in multiple timestamps was as important as an actor’s number of ties (i.e., degree centrality) or indirect ties (i.e., reach centrality). This perhaps could be another way of identifying leaders using SNA. Actors in all three categories appeared the same number of times. This suggests that simply being present in the network does not make one a leader.

#### Can early SNA data predict leaders?

##### Degree centrality

**Fig. 2:**
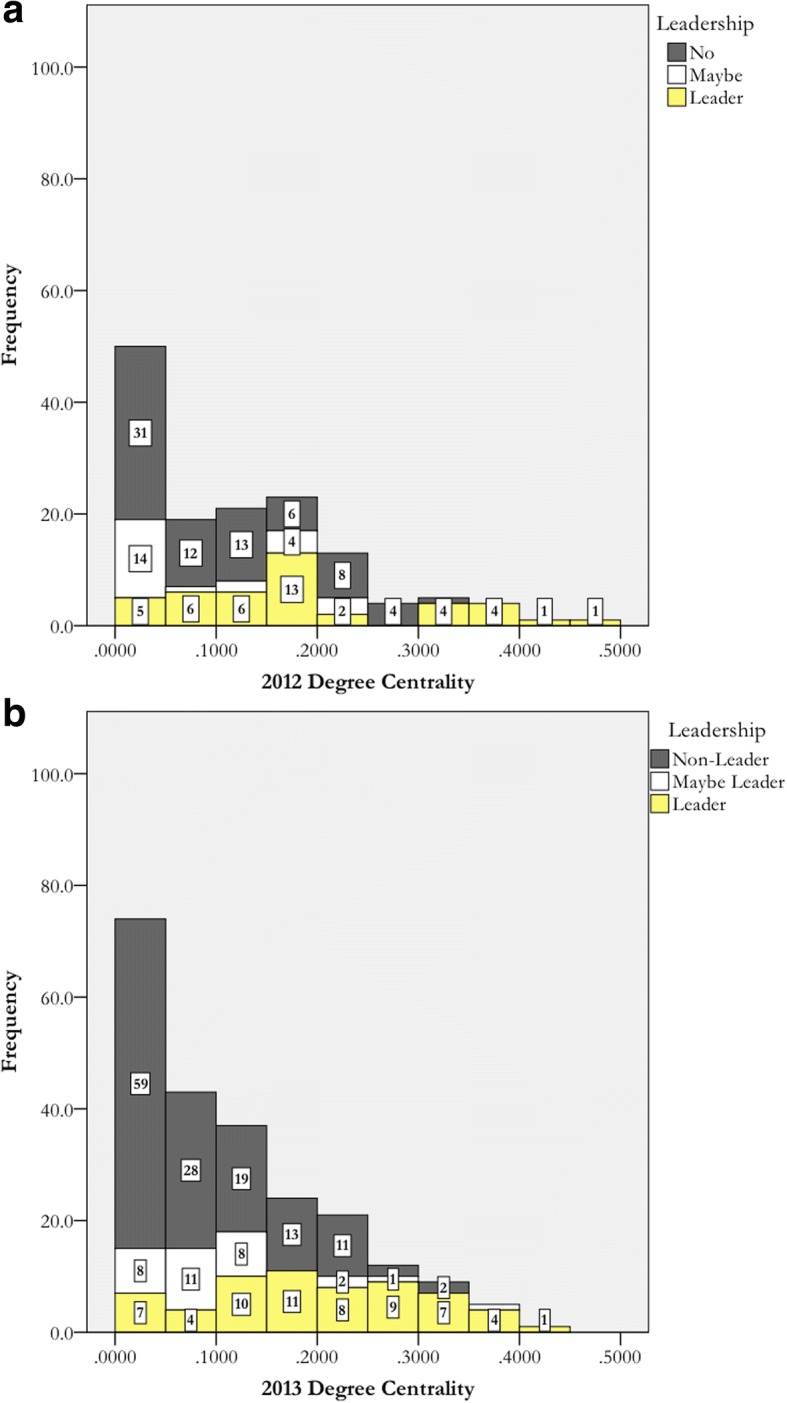
Histograms depicting the distribution of degree centralities and colored by leadership identity for 2012 (**a**) and 2013 (**b**)

While the results in Table 5 were promising and aligned with prior SNA research, we were interested in whether SNA could identify *individual* actors as leaders. We first analyzed the 2012 and 2013 data to see if these prior timestamps could be used to see who would be nominated as a leader in 2016. In Fig. 2, two histograms display the distribution of normalized degree centrality for 2012 and 2013. The width of each bar is 0.05.

Having high values for normalized degree centrality means that an actor is directly connected to most of the actors in the department. This would suggest that someone might be suitable to become a leader for these efforts. While the distribution is what we expected, that those with highest normalized degree centralities are clearly defined as leaders, we note that there were actors who did not follow this expectation. Those actors were considered either “false positives” or “false negatives” based on comparing the SNA data to the respondent-nominated leaders. These terms are defined as follows:

- *False SNA positive*: those who were not nominated as leaders on the survey but have higher than average centralities.
- *False SNA negative*: those who were nominated as leaders on the survey but have lower than average centralities.

To determine how many false SNA positives and false SNA negatives, a normalized degree centrality of 0.20, the approximate average normalized degree centrality for *Leaders* in 2012 and 2013 (see Table 5) was the cutoff point for identifying leaders using SNA. These data are displayed in Table 6. SNA identified 25 actors as leaders in the 2012 timestamp data. Had we just used SNA, approximately 70% of nominated leaders would not have been identified as leaders. Approximately half of the actors above the cutoff were not nominated as leaders. Had we just used SNA, they might have been selected as leaders even though they were not seen by their colleagues as leaders. The results are similar for 2013.

**Table 6:** Accuracy of leadership identification via degree and reach centralities

Year	Metric	False SNA negatives (% of total nominated leaders)	Leaders identified by both SNA data and nominations (% of total nominated leaders)	False SNA positives (% of leaders identified from SNA)
2012	Degree centrality	30 (71.4%)	12 (28.6%)	13 of 25 (52%)
	Reach centrality	21 (50.0%)	21 (50.0%)	17 of 38 (44.7%)
2013	Degree centrality	32 (52.5%)	29 (47.5%)	15 of 44 (34.1%)
	Reach centrality	19 (31.1%)	42 (68.9%)	39 of 81 (48.1%)
2016	Degree centrality	56 (67.5%)	27 (32.5%)	6 of 33 (18.2%)
	Reach centrality	30 (36.1%)	53 (63.9%)	16 of 69 (23.2%)

##### Reach centrality

**Fig. 3:**
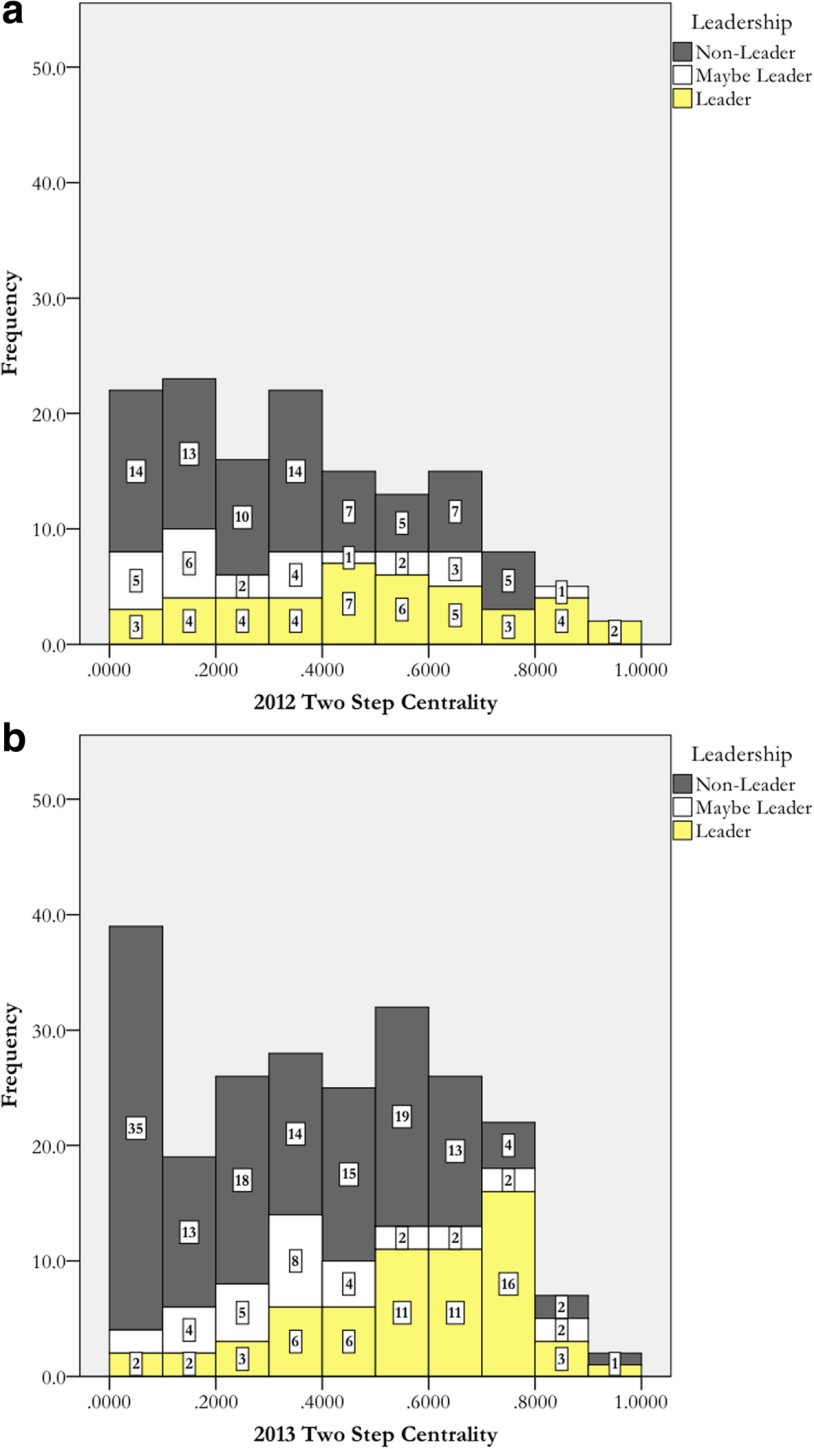
Histograms depicting the distribution of two-step centralities and colored by leadership identity for 2012 (**a**) and 2013 (**b**)

We did a similar analysis for two-step centrality for each of these years. Those histograms are displayed in Fig. 3. Each bin is 0.1 wide. For two-step reach centrality, high normalized values indicate that an actor is connected to others in their department either through direct ties (i.e., one step away) or friends of friends (i.e., two steps away). Similar to degree centrality, two-step centrality histograms display the expected distribution but with a number of false SNA positives and negatives. We used 0.50 as our cutoff, an approximation of the average two-step centrality for both the 2012 and 2013 data (see Table 5). These data are displayed in Table 6. Many nominated actors would not be identified as leaders with just SNA, and many actors who were not nominated would be identified as leaders.

#### Do recent SNA data provide better leadership identification?

##### Degree and reach centrality

**Fig. 4:**
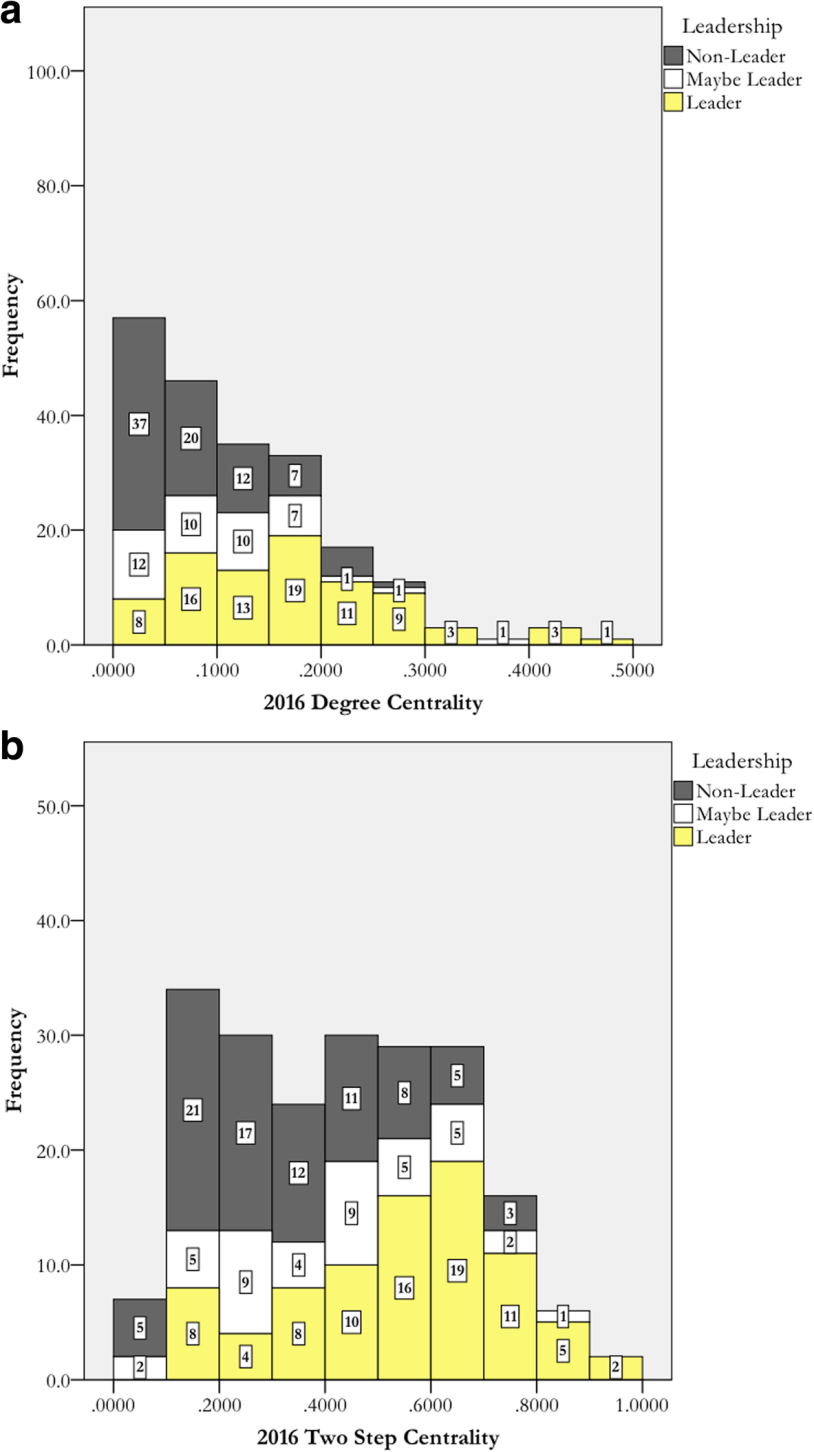
The 2016 distribution of degree centrality (**a**) and two-step centrality (**b**), with colors indicating leadership identity

The analysis in the prior section was repeated with the 2016 data. Our hypothesis was that SNA-identified leaders and the respondent-nominated leaders would be more congruent with more recent data. We used the same cutoff values, because the 2016 data were similar to 2012 and 2013. These data are displayed in Fig. 4. The false positive and false negative data are in Table 6. These histograms have fewer false SNA positives. However, there are still many false SNA negatives.

#### Are some leadership categories more prone to false negatives?

We checked if the false negatives issue was linked to one of the categories. These results are in Table 7. While many of the false SNA negatives are within the both leaders category, approximately half are not. We suspect that the low number of potential leaders as false SNA positives in 2012 and 2013 is due to those actors not being in the department (i.e., they were not working at the institution).

**Table 7:** False negatives broken down by leadership category

		Total false negatives from the SNA data	Current leaders	Potential leaders	Both leaders
2012	Degree centrality	30	12	3	15
	Reach centrality	21	7	2	12
2013	Degree centrality	32	9	4	19
	Reach centrality	19	5	3	11
2016	Degree centrality	56	12	22	22
	Reach centrality	30	3	15	12

#### Are respondents identifying leaders based on title, and does that lead to false SNA negatives or false SNA positives?

One concern regarding respondent-nominated leaders was that respondents would select those who have formal leadership titles (e.g., dean, department chair) and overlook those who are leading but lack a title. Perhaps the false SNA negative and false positive issues were due to title-identified leaders. Those with titles may not be as active in teaching discussion because they have other responsibilities and thus, this would lead to false negatives. Alternatively, those with formal titles could also not be considered leaders in STEM education change by their departments but still interact often regarding teaching as a result of their title, leading to false positives.

We examined the leaders category to see how many actors with formal leadership titles pertinent to education were also respondent-nominated leaders. These titles include: department chair, director of undergraduate studies, director of graduate studies, course coordinator, named leader for the change initiative, and membership to a committee related to education. Using each department’s website, we found 36 individuals with one of these titles. Twenty were identified as a leader*.* Four were considered maybe leader. All six chairs were nominated as some type of leader.

This result suggests that although title-designated leaders were often respondent-nominated leaders, merely having a title is not sufficient be nominated as a leader. Twelve individuals (33%) with relevant titles were not identified a leader or maybe leader by respondents; these twelve individuals also were not false negatives or false positives. Respondents also nominated many leaders without titles. Of the 93 leaders, sixty-nine (74%) do not have a formal leadership title.

## Conclusions

This study sought to examine whether teaching discussion social network data alone can be used to identify leaders for STEM education change initiatives. We found that SNA data yield a different set of leaders than respondent nomination. In the following sections, we discuss these results and their implications for using SNA to inform change initiatives.

### Discussion: can SNA metrics be used to predict or identify leaders in STEM education reform?

The SNA data do depict the expected distribution: many actors who are nominated as leaders in STEM education change work tend to have many direct ties (i.e., high degree centrality) as well as a high total of direct and “friends of friends” ties (i.e., high two-step reach centrality). Many actors who are not considered leaders tend to have lower metrics. However, this is not universally true. We saw a non-trivial number of false SNA negatives, those who were nominated as leaders by survey respondents but would not have been identified merely by SNA data. We also saw a number of false SNA positives, those who were not nominated as leaders by survey respondents but would have been identified as leaders based on SNA data.

Further analysis of the false SNA negatives did not yield much more insight. Within the three categories of leaders (current leaders, potential leaders, and both leaders), many of the false SNA negatives were in the both leaders category. Being categorized as both leaders may have been because respondents interacted less with those actors and thus were less sure how to categorize them. However, a non-trivial number were in the current leaders and potential leaders categories for each timestamp. Potential leaders may have had fewer ties because they were newer to change work or department. In the future, they may have enough ties to indicate they are leaders. However, it is curious why those perceived as current leaders would be false negatives. We assumed that actors who were perceived as current leaders would be active in teaching discussion.

We also considered whether respondents simply selected title-designated leaders. We thought this could have led to false SNA positives (e.g., their jobs required them to be active in teaching discussion, even if they were not considered leaders) or false negatives (e.g., those with titles do not have the time to be active in the teaching discussion network due to their job responsibilities). Title-designated leaders did not contribute to false SNA positives or false negatives. Some title-designated leaders were not nominated leaders.

Reflecting upon the data, we considered how response rates could impact the results. While we took care to use a technique to mitigate this issue (i.e., considering the data undirected), response rates might still impact the results. Recall it is possible that part of the network could be missing if there are no respondents from a section of the network. Perhaps had more respondents answered the survey, the false positives would have been identified as leaders by respondents or the false negatives would have higher centralities. Even with a nearly perfect response rate, it is also possible that the false positives and false negatives would still exist.

The response rate issue could be perceived as a flaw in this study. However, in Andrews et al. (2016) study that looked at similar teaching discussion networks, response rates from four departments ranged from 33 to 63%. Our response rates are comparable to theirs, suggesting this is not a unique issue to our study. The practical implication is that even if survey-generated teaching discussion SNA data with a perfect rate is suitable for identifying leaders, it may be unlikely to obtain a perfect response rate.

While these results may seem to contradict the SNA literature on leaders and our own findings that respondent-nominated leaders on average have higher metrics than non-leaders, we note that we are ultimately interested in identifying *individuals* as leaders. The SNA studies and our findings in Table 5 show averages. The details of individuals can get lost in averages. When using the data to select individuals, researchers should consider collecting and analyzing data so individuals can readily be found. In our case, plotting out the data in a histogram was useful to see degree and two-step reach centrality of individual actors.

Because selecting leaders is important to ensure the change initiative is successful, these data suggest that one should use multiple methods of identifying a leader as Valente and Pumpuang (2007) recommended. Based on the literature on changing teaching practices in STEM higher education, we know that change leaders should possess skills (e.g., understanding how to overcome barriers) and be well connected. If leaders in STEM education initiatives were selected simply by using network data, there is non-trivial chance that they would overlook a leader or select someone whose departmental members do not recognize as a leader. If leaders are selected simply based on respondent nomination, the leader may lack ties that are useful for spreading ideas or gaining new perspectives. Either scenario could hinder change efforts and threaten success.

Besides leadership identification, a similar study could be used as a tool for leadership planning. If nominated leaders are not well connected, those working on the change initiative could devise mechanisms to help connect nominated leaders. Likewise, there may be individuals who are well connected, interested in leading STEM education projects, and are not nominated as leaders. It may be useful to consider about why they are not nominated.

### Conclusions, recommendations, and further research

#### Conclusions

Our study looked at whether SNA alone can predict or identify leaders. We found that:

- Leaders *on average* have more ties and reach in their networks than non-leaders
- SNA yields a somewhat different set of leaders than respondent nominations.

Both methods have advantages. Nominations are useful for knowing whom departments trust for this work, and SNA is useful for determining who is well connected. Using just nominations could lead to selecting individuals who are poorly connected. Using just SNA could lead to selecting individuals who are not seen as leaders. Using multiple methods of identification, as we have done, would mitigate these issues.

#### Future research

The current study demonstrates some practical uses for studying leadership and networks within departments involved with a STEM education change initiative. There are several avenues for further research to deepen our understanding of leadership in this area. Areas include as follows:

- Determining more differences among current, potential, and both categories. There may be other aspects these data do not capture. This may be useful in understanding what experiences potential leaders need to transition to a current leader and why some leaders are not so distinctly defined.
- Investigating “false SNA positives” and “false SNA negatives.” It would be useful to know why some actors have many ties in the teaching discussion network but are not perceived as leaders. Similarly, it would be useful to know why some leaders have few teaching discussion ties.
- Studying how change initiatives impact how respondents nominate leaders. The presence of change initiatives may mean that leaders are more visible and readily identified by respondents. Approximately 30% of all actors in this study were considered a leader of some kind. Institutions that do not have a change initiative may have fewer nominated leaders. Similarly, institutions that have new change initiatives may have fewer nominated leaders. It also would be important to see whether leaders remain visible after the change initiative ends.
- Examining other types of relevant network data in a similar study. As noted earlier, teaching discussion networks are important for change in instruction (e.g., Sun et al. 2013). A teaching discussion network seemed suitable for identifying leaders because we hypothesized leaders would be more active in discussing teaching. However, there may be other networks in higher education that may be better suited for identifying leaders. One example could be studying which actors participate in which teaching activities (e.g., learning communities).

By continuing to expand our knowledge regarding how leadership operates in STEM education change initiatives, we can support the next generation of STEM education leaders and sustain or even grow current efforts.

## Footnotes

1 Unless otherwise noted, all error measurements are standard error of the mean.
